# Supplementary figures and images for: Forced Overexpression of Signal Transducer and Activator of Transcription 3 (STAT3) Activates Yes-Associated Protein (YAP) Expression and Increases the Invasion and Proliferation Abilities of Small Cell Lung Cancer (SCLC) Cells
Source: Biomedicines. 2022 Jul 14;10(7):1704. doi: 10.3390/biomedicines10071704 (PMC9313375; doi:10.3390/biomedicines10071704)

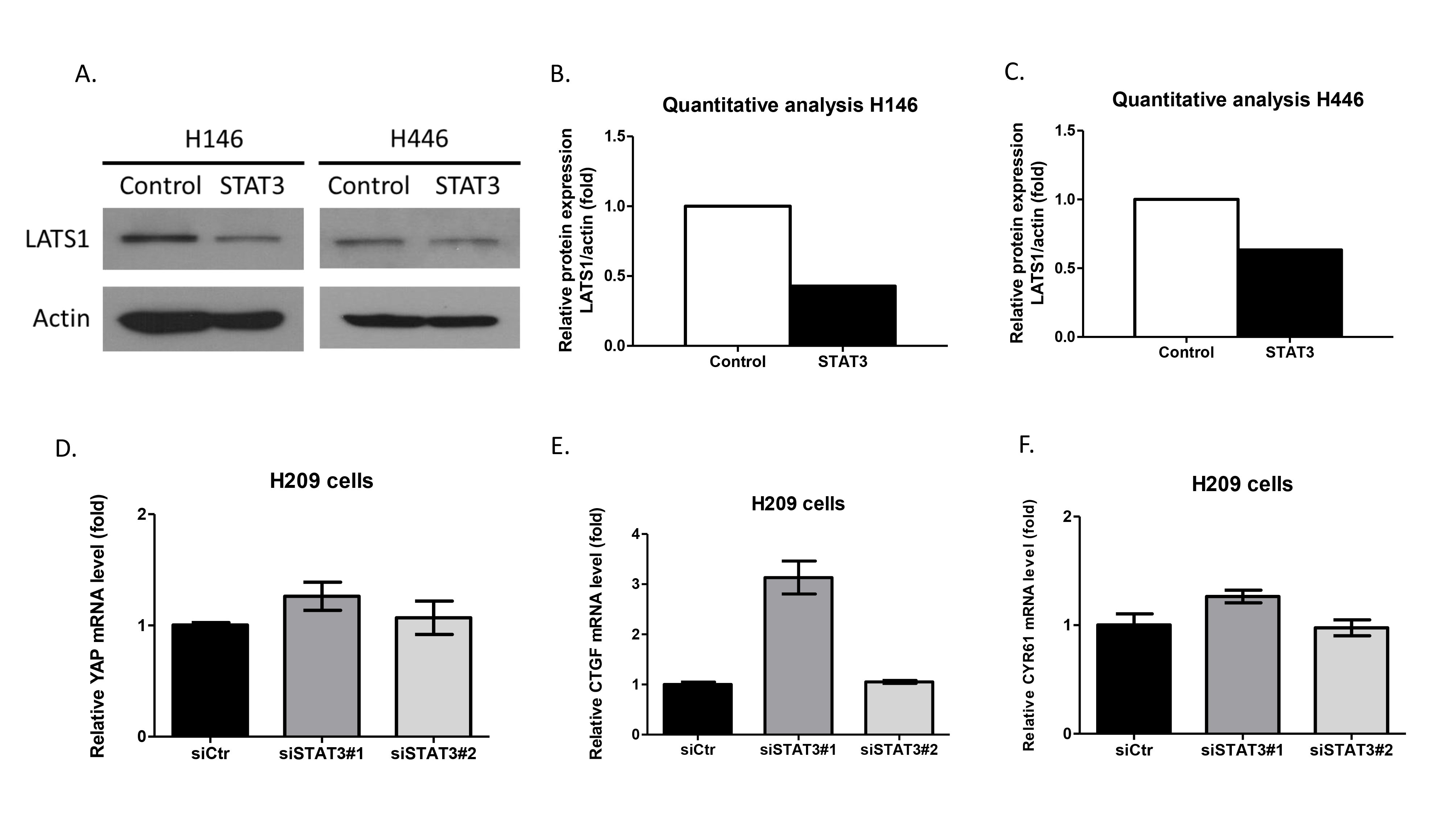

Supplement: Supplementary file 1 [file biomedicines-10-01704-s001.zip › biomedicines-1781866-supplementary.tif]
